# Methylation Profiling in Diffuse Gliomas: Diagnostic Value and Considerations

**DOI:** 10.3390/cancers14225679

**Published:** 2022-11-18

**Authors:** Anna Wenger, Helena Carén

**Affiliations:** 1 Sahlgrenska Center for Cancer Research, Department of Medical Biochemistry and Cell Biology, Institute of Biomedicine, Sahlgrenska Academy, University of Gothenburg, 413 90 Gothenburg, Sweden; 2 Wellcome Sanger Institute, Wellcome Genome Campus, Hinxton, Cambridge CB10 1SA, UK

**Keywords:** methylation, classification, diffuse gliomas, glioblastoma, intratumour, heterogeneity, calibrated score, *MGMT*

## Abstract

**Simple Summary:**

Diffuse gliomas are a type of brain tumour afflicting adults and children, often with a fatal outcome. An accurate diagnosis is required to give patients correct treatment and for the allocation of homogeneous diagnoses into clinical trials. Diagnosis is historically based upon the visual appearance of the tumour in a microscope, but is nowadays also complemented by molecular analyses of how the tumour cells’ DNA has altered. One aspect that has gained considerable interest is the so-called DNA methylation pattern of the tumour. The pattern is a sort of bar code for the tumour, and this bar code differs for various tumour types. As such, the methylation pattern can be used for the diagnosis of tumours and stratification into methylation subclasses. Here, we review the utility of DNA methylation as a diagnostic tool in diffuse gliomas and considerations for its use.

**Abstract:**

Diffuse gliomas cause significant morbidity across all age groups, despite decades of intensive research efforts. Here, we review the differences in diffuse gliomas in adults and children, as well as the World Health Organisation (WHO) 2021 classification of these tumours. We explain how DNA methylation-based classification works and list the methylation-based tumour types and subclasses for adult and paediatric diffuse gliomas. The benefits and utility of methylation-based classification in diffuse gliomas demonstrated to date are described. This entails the identification of novel tumour types/subclasses, patient stratification and targeted treatment/clinical management, and alterations in the clinical diagnosis in favour of the methylation-based over the histopathological diagnosis. Finally, we address several considerations regarding the use of DNA methylation profiling as a diagnostic tool, e.g., the threshold of the classifier, the calibrated score, tumour cell content and intratumour heterogeneity.

## 1. Introduction

Diffuse gliomas are of glial (astrocytes, ependymal cells, and oligodendrocytes) origin and, as the name implies, characterized by a diffuse tumour growth pattern in the brain. The diffuse and infiltrative nature of the tumour poses significant challenges, as it severely compromises complete tumour resection. Further, tumour heterogeneity in terms of treatment response, genetic alterations and subclones with varying sensitivity to chemo- and radiotherapy are also considered to be factors in treatment failure and tumour relapse [1,2,3], in many cases leading to the death of the patient. Despite decades of intense research, diffuse gliomas still cause significant morbidity in adults and children [4]. While diffuse gliomas were previously thought to be the same disease regardless of age, it has become obvious in recent years that they differ significantly in childhood vs. adulthood [5,6,7]. Gliomas in adults often have mutations in genes encoding isocitrate dehydrogenases (IDH), and these mutations are rare in children [5]. Similarly, histone 3 (H3) mutations frequently occur in children, and are less common in adults [6,8,9]. These differences were recognized in the newest edition (2021) of the CNS (central nervous system) tumours World Health Organisation (WHO) classification, where they are now divided into adult-type and paediatric-type gliomas [9].

The classification of brain tumours has historically been solely based on histopathological features of the tumours [10], whereas the latest editions incorporate molecular markers (e.g., IDH mutation, 1p/19q codeletion etcetera) and DNA methylation profiles [9,11,12]. Methylation mainly occur on so-called CpG sites (cytosine DNA base followed by a guanine base), of which we have around 30 million in the human genome [13]. The methylation status of these CpG sites make up the methylation profile, a “barcode” of the tumour and, importantly, this profile is altered in tumours compared to healthy tissue [14,15,16]. Further, the methylation profile differs between various kinds of brain tumours, and multiple classifiers have been created for tumour classification purposes [12,17,18] and implemented in clinical practice for certain tumours/age groups in several countries. This review article will: (1) provide an overview of the differences between adult and paediatric gliomas, (2) examine the utility of DNA methylation as a novel diagnostic tool, (3) examine the considerations for its use, and (4) study how it is affected by the heterogeneity in diffuse gliomas.

## 2. Adult vs. Paediatric Gliomas

The frequency of cancer drastically increases with age, from 18 cases per 100,000 in children (0–18 years) to 2000 cases per 100,000 in adults over 65 years of age [19]. At each cell division, 2–10 random mutations are generated [20] and a longer life thus leads to more accumulated mutations and a higher risk that one mutation will affect genes involved in tumour development (e.g., activate oncogenes/inactivate tumour suppressor genes). Accordingly, tumours in adults have many more mutations and chromosomal copy-number alterations (CNA) than childhood tumours [7,21]. Some childhood tumours, such as pilocytic astrocytoma (a circumscribed astrocytic glioma), have a “silent” genome with few or even no CNA [21]. The mutations in childhood tumours generally vary from adulthood cancer as they are frequently targeting epigenetic and developmental regulation [6,22,23]. Consequently, the most common types of cancer differ between adults and children. Breast, prostate and lung cancer are the three leading types in adults whereas the most common in children are leukaemia, CNS tumours and lymphoma [24].

CNS tumours in children constitute 27% of all cancer cases whereas in adults only 1% [4,24]. The most common types of gliomas also differ between the age groups; glioblastoma is most common in adults whereas it is quite rare in children [4] (note that the diagnosis glioblastoma is not present for children in the CNS WHO 2021 [9] edition). Unfortunately, the prognosis is dismal in all age groups [4]. Another separator between the adult and paediatric gliomas are the type of mutations; IDH mutations are, e.g., rare in children while common in adults [5]. H3 mutations were originally thought to be unique to paediatric tumours [6,25], but have subsequently been detected in adults as well, albeit at a low frequency [8].

## 3. CNS WHO Classification of Diffuse Gliomas

The sequencing efforts over the last decades have revealed major differences between adult and paediatric tumours and the current CNS WHO 2021 classification of gliomas reflects this, as tumours are separated into adult-type and paediatric-type [9]. We will here focus on the categories adult-type diffuse gliomas, paediatric-type diffuse low-grade gliomas, and paediatric-type diffuse high-grade gliomas, while circumscribed astrocytic gliomas, glioneuronal and neuronal tumours, and ependymal tumours are described elsewhere.

Adult-type diffuse gliomas are mainly diagnosed based on IDH mutation, which is more frequent in lower-grade gliomas [26]. An IDH wildtype adult-type diffuse glioma is diagnosed as a glioblastoma (CNS WHO grade 4), and they frequently have *telomerase reverse transcriptase (TERT*) promoter mutations, chr7 gain and chr10 loss and/or EGFR (epidermal growth factor receptor) amplification [9,27]. One of these alterations in a diffuse astrocytoma IDH wildtype is enough to classify it as a glioblastoma even if histological high-grade features are lacking [9]. This criterion is new for the 2021 classification and it was established as several studies reported that diffuse astrocytoma IDH wildtype with the histological grade II-III (according to CNS WHO 2016) had the clinical behavior of glioblastoma when they had one or more of these alterations [28,29]. Diffuse astrocytoma IDH wildtype without these molecular features are rare and are not included as a tumour type in CNS WHO 2021 [9,30]. Paediatric-type gliomas (described below) should instead be considered as a diagnosis for these tumours, especially in younger adults.

The IDH mutant diffuse gliomas are separated based on 1p/19q codeletion [9], in which case they are diagnosed as oligodendrogliomas (grade 2–3), and otherwise as astrocytomas (grade 2–4). The astrocytomas commonly have loss of α-thalassemia mental retardation X-linked (ATRX), which is retained in oligodendrogliomas, and astrocytomas frequently have *TERT* promoter mutations. The grading is carried out on a scale of 1 to 4 and is based on an integrated assessment of histological features and biomarkers [9]. For instance, a homozygous deletion of CDKN2A/B in an IDH mutant diffuse astrocytoma automatically yields a grade 4 (the highest/most malignant grade), even if the histology is indicative of a low-grade tumour. It is important to note that the CNS grading is based on the aggressiveness of the tumour, and does not necessarily reflect the prognosis after treatment, e.g., a grade 4 tumour, such as a wingless (WNT)-activated medulloblastoma, can have effective treatment and a good prognosis; however, left untreated, the survival is poor [31]. A new addition to the CNS WHO 2021 classification is that the CNS grading is performed within types instead of between types. Previously, a grade 3 CNS tumour, regardless of the type, was expected to have a similar clinical behavior. In addition, the type of tumour automatically decided the grade of the tumour and there was no way to assign various grades to indicate different malignancies within tumour types. That is now possible with the CNS WHO 2021, as tumours are graded in relation to other tumours of the same type (within types) rather than other types.

Paediatric-type diffuse high-grade gliomas include four types: two of them are defined by different mutations in histone 3; diffuse midline glioma H3K27-altered, and diffuse hemispheric glioma H3 G34 mutant [9]. The third category consists of tumours that are both H3 wildtype and IDH wildtype (diffuse paediatric-type high-grade glioma, H3-wildtype and IDH-wildtype), whereas the fourth type occurs in infants and newborn babies (infant-type hemispheric glioma). It is notable that the diagnosis glioblastoma is no longer used for children, and the high-grade diffuse intrinsic pontine glioma (DIPG; frequently, but not always, with H3 mutations) included in earlier CNS WHO editions has also been omitted in favour of clear groups defined by the presence or lack of H3 mutations.

Paediatric-type diffuse low-grade gliomas contain four diagnoses [9] with similar histological appearances and they are, therefore, characterized by clear molecular features instead. The categories are; (1) diffuse astrocytoma MYB (MYB proto-oncogene, transcription factor)- or MYB proto-oncogene-like 1 (MYBL1)-altered, (2) angiocentric glioma (characterised by MYB alterations), (3) polymorphous low-grade neuroepithelial tumour of the young (PLNTY; abnormalities in BRAF (B-Raf, proto-oncogene, serine/threonine kinase) and fibroblast growth factor receptor (FGFR) family), and (4) diffuse low-grade glioma, mitogen-activated protein kinase (MAPK) pathway-altered (FGFR1, BRAF). PLNTY is a novel tumour type in children and young adults with an oligodendroglioma-like component (but lacking the characteristic 1p/19q codeletion and IDH mutation), delineated by its methylation pattern [32]. The tumour is also associated with a history of epilepsy [32].

## 4. DNA Methylation and How Methylation-Based Classification Works

DNA methylation is, in essence, the addition of a methyl group (CH_3_) to a cytosine, almost exclusively occurring on a CpG site [33,34]. It is a stable epigenetic mark that plays a crucial role in embryogenesis, development, X-chromosome inactivation, aging, cell growth and cell differentiation [35,36,37]. Methylation regulates gene expression where a methylated promoter region silences gene expression, whereas an unmethylated promoter allows for gene transcription [13,38]. This mechanism is involved in cancer development, where key tumour-suppressor genes are hypermethylated (silenced) and oncogenes hypomethylated (activated) [38,39]. A global hypomethylation is also common in many cancer types, and leads to chromosomal instability and an increase in mutations [40,41,42]. Another example of alterations in the methylation pattern in cancer is found in adult-type lower-grade gliomas with IDH mutations [43]. The IDH mutation leads to the production of the oncometabolite 2-hydroxyglutarate (2HG) and subsequent alterations in metabolic activity and genome-wide hypermethylation of CpG islands [5,43]. This was previously detected in colorectal cancer and dubbed the CpG island methylator phenotype (CIMP) [44], and this phenotype in lower-grade gliomas was thus called glioma-CIMP (G-CIMP) [45].

The cancer alterations described above, in combination with the methylation pattern reflecting the cell of origin [46], allows for tumour classification and subtyping. Several methylation-based classifiers for CNS tumours have been developed [12,17,18,47]. In this review, we will focus on the Molecular Neuropathology classifier (MNP; https://www.molecularneuropathology.org/mnp/classifiers/11; accessed on 11 November 2022) [12], which is the most common and has been integrated in clinical routine in several countries [8,48,49,50,51]. The MNP classifier used a reference cohort of almost 3000 samples of various kinds of CNS tumours to define tumour types with distinct methylation profiles. The most informative CpG sites for each type was selected and used to train the classifier through a random-forest algorithm, which uses multiple decision trees (“classification trees”) of the selected CpG sites to classify unknown samples into a tumour type. The combined score from the decision trees were recalibrated to span between zero and one and were intended to reflect the probability of an accurate classification. This score was referred to as the calibrated score, where the optimal trade-off between specificity and sensitivity (so-called Youden index) was reached at 0.84, while maximum specificity was reached at 0.96. The threshold was, therefore, set in the middle at 0.9 for tumour classes. Subclasses received a threshold of 0.5. Since the MNP classifier was published in 2018 [12], there have been updated versions of the classifier containing novel tumour types, more classification levels and different thresholds. Here, we focus on the newest 12.5 version of the classifier, which is currently unpublished. Compared to earlier versions, it has four classification levels and uses a 0.9 threshold on all levels. The levels are; a superfamily, a family, a class, and a subclass (Table 1). The methylation level most similar to the CNS WHO 2021 classification [9] varies; e.g., the methylation family glioblastoma IDH-wildtype is the best match to the CNS WHO classification in case 1, whereas the methylation subclass is most similar to the CNS WHO oligodendroglioma type in case 2. Note that multiple subclasses are not available for all classes (e.g., case 1), whereas the RTK1 subtype (class in case 3) has three subclasses (A, B and C).

## 5. Methylation-Based Classification in Diffuse Gliomas and Its Value

The benefits of methylation profiling in diffuse gliomas are summarized in the following sections and Table 2. One of the first tumours advanced by methylation-based classification was the paediatric brain tumour medulloblastoma. Methylation profiling identified four novel molecular subgroups (WNT, SHH (sonic hedgehog), Group 3 and Group 4), which differ in prognosis and, hence, receive treatment based on their subgroup [4,31,52,53,54]. The value of methylation profiling as a complement to histology has subsequently been demonstrated in several studies. Jaunmuktane et al. evaluated methylation profiling in adult brain tumours (including diffuse gliomas) and found that the diagnosis was changed in 25%, refined in 48% and confirmed in 25% of cases [8]. They also specifically examined 44 IDH wildtype diffuse gliomas of low-grade morphology with methylation profiling, and 41% were classified as tumours with high-grade behaviors such as glioblastoma, while the remaining 59% received a classification of various low-grade glial or glioneuronal types. It is important to note that methylation-based classification is user-independent, whereas inter- and intra-observer variability exists for histopathological evaluations [55]. In a separate study, methylation-based classification revealed that 16 of 166 (10%) of diffuse lower-grade gliomas (CNS WHO 2016) were classified as glioblastoma IDH wildtype [56]. Studies including paediatric patients in CNS tumour cohorts (all types, not only diffuse gliomas) also reported that the diagnosis was confirmed in around 40%, refined in 13–25%, and led to a change in diagnosis in 6–10% of patients, resulting in changed clinical management in 5% of the children [49,50,51]. These studies highlight the impact of methylation profiling in clinical routine for the diagnosis and treatment of paediatric CNS patients and have also been incorporated in the clinic in several countries.

Although glioblastoma methylation subclasses are not used clinically, a recent study reported that a survival benefit was seen in glioblastomas with maximised extent of resection in receptor tyrosine kinase (RTK) 1 and RTK2 methylation subclasses, but not in the mesenchymal subclass [57]. This questions the use of maximal resection in the mesenchymal subclass, requiring that the methylation subclass is known beforehand, and might be particularly useful at recurrence based on a mesenchymal subclass of the primary glioblastoma (assuming that the dominating methylation subclass remains at relapse). Another study similarly reported that patients with the RTK2 subclass of glioblastoma were more prone to developing seizures, thus providing information for postoperative control measures [58].

Another benefit of methylation profiling is that it can be used to generate CNA plots [48,59], which can be used to detect amplifications/deletions of diagnostic value, e.g., the 1p/19q codeletion in oligodendrogliomas. While methylation profiling cannot detect mutations per se, it detects the widespread methylation alterations occurring because of the mutation, and can thus classify tumours as IDH or H3 mutated/wildtype. The *O6-methylguanine-DNA methyltransferase* (*MGMT)* methylation status can also be predicted from methylation arrays, as described previously [60,61]. Further, methylation profiling can be carried out on DNA extracted from formalin-fixed paraffin-embedded (FFPE) tumours, which is the gold standard in clinical routine [48,54,62]. In addition, a low amount of DNA is required, making it a cost-effective analysis yielding the methylation profile and CNA from, e.g., tissue biopsies with limited material.

**Figure 1:**
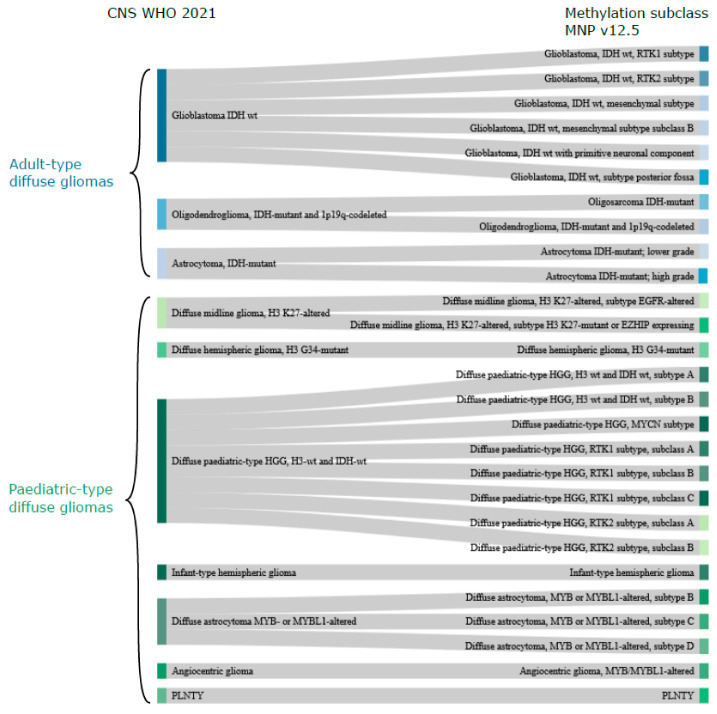
**CNS WHO 2021 vs. methylation-based classification**. Sankey plot illustrating the relationship between CNS WHO 2021 classification (left) of diffuse gliomas with the MNP methylation subclasses (classifier v12.5; right) based on the description and naming of the methylation subclasses (https://www.molecularneuropathology.org/mnp/classifiers/11; accessed on 11 November 2022). Note that adult-type gliomas (blue colours for the different tumour types) are most common in adults, but may occur less frequently in children and, likewise, that paediatric-type gliomas (shown in green colours for the various types) may occur in adults. There is no methylation subclass directly corresponding to the CNS WHO diffuse low-grade glioma, MAPK pathway-altered category and this is, therefore, not included in the plot. Abbreviations: wt—wildtype, HGG—high-grade glioma. PLNTY—polymorphous low-grade neuroepithelial tumour of the young, EZHIP—enhancer of zest homologs inhibitory protein, MYCN—MYCN proto-oncogene, bHLH transcription factor.

The methylation-based classification of diffuse gliomas, as expected, does not completely correlate with the CNS WHO classification. There are, however, many similarities, and the methylation superfamilies are termed similarly to the CNS WHO 2021 classification (adult-type diffuse gliomas, paediatric-type diffuse high-grade gliomas, etc.; Table 1). One of the main differences is that methylation-based classification further stratifies samples into multiple classes/subclasses, delineated by their methylation profile, as described below (Figure 1).

Glioblastoma in adults is, according to CNS WHO 2021 [9], “only” a glioblastoma, whereas the MNP classifier [12] v12.5 suggests six subclasses with distinct methylation profiles; RTK1, RTK2, mesenchymal subtype, mesenchymal subtype subclass B, primitive neuronal compartment, and subtype posterior fossa. The paediatric-type high-grade gliomas also have more methylation subclasses (*n* = 12) compared to the four types (H3 K27, H3 G34-mutant, H3 wt and IDH wt, infantile hemispheric glioma) included in the CNS WHO 2021 classification (Figure 1). Adult-type IDH mutant gliomas, on the other hand, have methylation subclasses corresponding to the types in CNS WHO 2021. The exception is the novel methylation subclass Oligosarcoma IDH mutant, which contains a mix of oligodendroglial and sarcomatous features [63]. Paediatric-type, diffuse, low-grade gliomas have four types in CNS WHO 2021 [9], and angiocentric glioma has a corresponding methylation subclass, as does PLNTY. Then, there are three subclasses of diffuse astrocytoma, MYB or MYBL1-altered, whose methylation profiles are most similar to paediatric MYB/MYBL1-altered diffuse astrocytomas and angiocentric gliomas [64].

The higher number of methylation subclasses compared to CNS WHO diagnoses highlights the potential of methylation classification for further patient/tumour stratification. On several occasions, subclasses identified by methylation profiling have later been introduced into the CNS WHO classification (e.g., for medulloblastoma [52,65] and ependymoma [66,67]). The methylation subclasses also provide an opportunity for treatment to be targeted to specific subclasses. The RTK subclasses of glioblastoma and paediatric-type high-grade gliomas are examples of this, as several RTK inhibitors exist [68,69], but the glioblastoma methylation subclass is currently not used for treatment decisions. The methylation subclasses will hopefully be further integrated into the CNS WHO diagnostic categories, as well as being used to inform treatment in the upcoming years. If not, we must ask whether the methylation subclasses are meaningful to the clinic, and if additional subclasses and further subtypes within the subclasses provide any value.

## 6. Considerations Regarding Methylation-Based Classification

Methylation-based classification, as a diagnostic tool, is relatively new and we will cover some limitations, challenges and considerations regarding its interpretation and clinical use. One of the main considerations is the calibrated score and the threshold used. As mentioned above, previous versions of the MNP classifier [12] had a 0.5 threshold for subclasses and an official 0.9 threshold for the class, although the alternative threshold of 0.84 (Youden index) was frequently employed in studies with clinical samples [8,48,49,56]. It should be noted that the MNP classifier was designed as a research tool, not for diagnostic purposes, but is increasingly implemented as such, given the demonstrated benefits of its use (as discussed above).

The new classifier version 12.5 also has an official 0.9 threshold, which begs the question of whether a 0.84 threshold can be employed here as well. Until the data behind the new version and the accompanying specificity and sensitivity scores have been published, we do not know the answer. The threshold, whether 0.9 or 0.84, can in itself be viewed as problematic, as it implies that anything below the threshold is of no use (Table 3). Biology is rarely black and white and, just like a carton of milk is usually good after its expiration date; in our experience, classifications with calibrated scores slightly below the threshold are usually fine. However, the milk will slowly go bad and one day be unusable. The question is, therefore, how low the calibrated score can go and remain usable. Scores below 0.3 are considered “not classified” and the CNS WHO 2021 [9] states that scores below 0.5 “should probably be discarded”. Where between 0.5 and 0.9 the “unusability” score lies is unclear, and may even vary for different tumour types. What we do know is that these scores can be useful, but should be integrated with the histological features of the tumour for the final diagnosis.

Several factors, such as lower tumour cell content, poor DNA quality and tumour heterogeneity (discussed further below), can lead to lower calibrated scores [48,49]. A low tumour cell content, which is a risk in infiltrative tumours such as diffuse gliomas, may even lead to an incorrect classification of “control tissue” by the MNP classifier (Table 3). The tumour cell content can often be improved through fluorescence-guided surgery (e.g., 5-aminolevulinic acid), or by macrodissection of specific areas for the methylation profiling. “Control tissue” classifications are not frequent and more commonly, the methylation-based classification is in line with the histopathological diagnosis, but with a calibrated score below 0.9, as the methylation pattern is skewed by the contribution of non-cancer tissue. Alternatively, the methylation-based classification is non-contributory. We experienced e.g., a paediatric glioblastoma [70] by histopathological analysis (CNS WHO 2016), which was similarly classified by methylation (paediatric-type diffuse high-grade glioma) for the primary tumour. The relapse tumour did not match a methylation superfamily, but was weakly assigned (calibrated score 0.36) as a low-grade glioma, which was surprising. However, the relapse tumour was evaluated by histology to 10% tumour cell content and had no CNA, whereas the primary had several. In this case, there was a high degree of normal tissue in the relapse sample, which contributed to the methylation-based classification.

A tumour cell content of >70% was recommended for the MNP classifier [48], but we found that diffuse gliomas with a tumour cell content as low as 40–50% could still be successfully classified (calibrated score > 0.9) [49,70]. Further, certain diffuse low-grade gliomas have a relatively low innate tumour cell content and cannot be profiled by methylation if adhering to the >70% recommendation. We therefore advise that samples below <70% tumour cell content can be run with the MNP classifier, but that the classification, calibrated score and CNA need to be evaluated for feasibility in the context of the tumour cell content and the histological analysis.

## 7. Heterogeneity in Diffuse Gliomas

Diffuse gliomas are a heterogeneous group with large differences between patients and within individual tumours. Cancer stem cells (CSC), also called tumour-initiating cells or glioma stem cells, have been shown in adult glioblastoma and paediatric-type high-grade gliomas [71,72,73,74,75,76]. These cells are believed to initiate tumourigenesis and lead to tumour relapse, as they are more resistant to radiotherapy and chemotherapy [74,75]. This is, in part, due to their slower cycling rate, plasticity and ability to enter a quiescent state [77,78]. These qualities allow them to escape therapies targeting dividing cells (e.g., chemotherapy) or certain cell states. According to the CSC model, CSC are able to self-renew and produce progeny of various differentiation states in a hierarchical manner [79,80]. The stochastic model of tumour heterogeneity, on the other hand, states that a normal cell will acquire tumourigenic properties through genetic/epigenetic alterations, leading to a growth advantage [81]. This cell will subsequently gain further alterations and the clone that is best equipped to grow will dominate the population until another clone with additional alterations/growth advantages takes over, and so forth (survival of the fittest).

Intratumour heterogeneity has been demonstrated in paediatric-type high-grade gliomas in terms of mutations, CNA, histopathology, gene expression and multitude of subclones [82,83]. Similarly, adult glioblastoma are heterogeneous on a regional as well as single-cell level regarding genetic alterations and transcriptional expression [84,85,86]. The treatment response also varies due to multiple subclones and differentiation states [2,3]. Here, we will discuss methylation patterns and methylation-based classification in spatially separated regions of adult-type and paediatric-type diffuse gliomas, starting with adult glioblastoma.

Several studies have sampled specimen from different regions of the same glioblastoma tumour and profiled them using methylation-based classification [87,88,89,90]. We showed that 5 of 12 (42%) glioblastoma tumours had different methylation subclasses within their tumours [87]. This subclass heterogeneity was determined with the newest version of the MNP classifier at the time and its suggested threshold (v11.b4; subclass threshold = 0.5) [12]. A subsequent study by Verburg et al. used the same version of the MNP classifier and found subclass heterogeneity in 5 of 11 (45%) glioblastoma tumours [88]. A recent study in 2022 used the methylation-based glioma classifier by Ceccarelli et al. [18], and detected subclass heterogeneity in 22 of 56 (39%) glioblastoma patients [90]. All three individual studies, using two types of classifiers, report subclass heterogeneity in around 40% of glioblastoma patients, suggesting a relatively high intratumour methylation heterogeneity in glioblastoma. While the glioblastoma methylation subclasses are not in clinical use, it is possible that they will be in the future, either in refined and more distinct subclasses or in their current form. For the latter scenario, the subclass heterogeneity needs to be considered.

We and others have noted that the glioblastoma subclasses RTK2 and mesenchymal are often seen in the same tumours [12,87], suggesting either a frequent co-occurrence of these subclasses, or a similar biology and the potential need for refinement. The mesenchymal subclass occasionally coincided with the lowest tumour cell content [87], indicating that this subclass potentially represents tumours with a lower tumour cell content. We analysed a cohort of glioblastoma tumours using the newest v12.5 of the MNP classifier and found subclass heterogeneity in 3 out of 10 patients (30%) using the same threshold (0.5) as the older version [89]. Applying the 0.9 threshold recommended for the 12.5 version yielded no patients with subclass heterogeneity. Further studies with larger cohorts are required to determine if subclass heterogeneity in glioblastoma also occurs using the new version, and which threshold that can be applied.

The number of CpG sites with differing methylation values within glioblastoma tumours have also been investigated in two of the studies referenced above [87,88]. These sites are frequently referred to as differentially methylated positions (DMP; a position is one CpG site on the methylation array). The impact of methylation alterations at single CpG sites may seem trivial, but they have the potential to alter the methylation state of biomarkers (see description of *MGMT* below) and switch the methylation subclass, as relatively few CpG sites define the subclasses. The studies used different thresholds for DMP calling, but both showed that a relatively low number of CpG sites are responsible for the observed intratumour heterogeneity [87,88]. Verburg et al. also showed that the number of DMP increased with increasing physical distance of samples within the same tumour [88]. The DMP were rarely shared between patients, suggesting that they occur randomly, but were shown to preferentially occur within so-called OpenSea regions [87]. This could potentially be because alterations more easily occur in these regions compared to the more evolutionary conserved CpG islands.

IDH mutant diffuse gliomas have been reported as homogeneous in terms of intratumour subclasses by us with v12.5 (7 patients) [89], whereas Verburg et al. demonstrated that 3 of 16 (19%) patients had subclass heterogeneity with the v11.b4 classifier [88]. In all cases, the subclass switch was between “IDH glioma astrocytoma” and “IDH glioma high-grade astrocytoma”. The low subclass heterogeneity could be explained by the fact that relatively few methylation subclasses exist for these tumours; in addition to the two astrocytoma subclasses, there are only oligodendrogliomas and oligosarcomas. Furthermore, the subclasses are clearly defined, e.g., the oligodendrogliomas have 1p/19q codeletion, whereas the astrocytomas do not. DMP were demonstrated in IDH mutant diffuse gliomas as well, but there were fewer than in grade 4 glioblastomas [87,88,89]. This suggests that the number of methylation alterations within a tumour increases in more high-grade tumours.

Studies on intratumour methylation heterogeneity in paediatric-type diffuse gliomas are, unfortunately, rare. We performed a study with several types of CNS tumours in children, where a few diffuse gliomas were included (no high-grade) [70]. The results showed that all tumour types had homogeneous intratumoural subclasses (MNP classifier v12.5, threshold 0.9) [70], which is promising for clinical diagnostics. There were, however, indications of subclass heterogeneity (calibrated score below 0.9). The DMP showed a similar pattern to the adult glioblastoma with alterations randomly located in less conserved regions.

In addition to methylation-based classifiers, there are also methylation-based biomarkers, with the most well-known arguably being *MGMT*. MGMT is a DNA repair enzyme, and it counteracts the damage in the DNA caused by the alkylating agent temozolomide [91], the standard chemotherapy used for glioblastoma [92]. A methylated *MGMT* promoter silences the *MGMT* expression [93] and patients with methylated *MGMT,* therefore, have a better prognosis compared to those with an unmethylated *MGMT* promoter [94]. *MGMT* methylation is clinically used for treatment decision in elderly glioblastoma patients [95]. Patients with methylated *MGMT* receive temozolomide and radiation, whereas patients with an unmethylated *MGMT* receive radiation only and are thus spared the harsh temozolomide treatment, as there is little added benefit when MGMT is active. We [87], and others [90,96,97], have demonstrated that the *MGMT* methylation status differs spatially within glioblastoma tumours. It means that patients could receive a different treatment depending on the region of the tumour that was sampled. This raises concerns about the use of *MGMT* as a biomarker and warrants further studies. Similarly, we have also shown that the proposed biomarker methylation age [98,99,100] differs spatially within glioblastoma, oligodendrogliomas and astrocytomas [87,89].

## 8. Conclusions and Future Directions

DNA methylation profiling is undoubtedly a powerful diagnostic tool in diffuse gliomas and has already influenced the CNS WHO classification. As it is incorporated further into clinical practice, clinical studies and research studies, it is important to take into account the considerations reviewed here; e.g., calibrated scores below the official threshold can be used (incorporated with histopathological analysis), but how low scores can be trusted? How many methylation subclasses are needed and meaningful for patient stratification regarding prognosis, treatment and clinical management? In the extreme scenario, one could say that every patient represents their own methylation subclass, but there is, of course, no point in endlessly creating further subclasses if they eventually do not benefit the patient (targeted treatment/management) or provide biologically relevant subclasses.

Heterogeneous methylation subclasses within the same tumour, as addressed here, also need to be addressed if the subclasses are to be used for treatment decision. *MGMT* is already in clinical use for elderly glioblastoma patients, despite several reports of intratumour heterogeneity [87,90,96,97] and uncertainty regarding the optimal threshold [101,102,103]. Methylation profiling, as a diagnostic tool, will likely extend to other tumour types as well. There is, e.g., a methylation-based sarcoma classifier available [104]. As methylation classification develops, novel diffuse glioma types and subclasses will be identified and subsequently validated for their utility through clinical use and by the large brain-tumour research community. This will result in an even more accurate and robust classifier, benefitting adults and children with diffuse gliomas.

## Figures and Tables

**Table 1:** The four levels of hierarchy according to v12.5 of the MNP classifier for three cases with different types of diffuse gliomas. RTK—receptor tyrosine kinase, wt—wildtype.

Hierarchy	Case 1	Case 2	Case 3
Superfamily	Adult-type diffuse gliomas	Adult-type diffuse gliomas	Paediatric-type diffuse high-grade gliomas
Family	Glioblastoma, IDH-wt	Diffuse glioma, IDH mutant	Diffuse paediatric-type high-grade glioma, H3-wt and IDH-wt
Class	Glioblastoma, IDH-wt, RTK1 type	Diffuse glioma, IDH-mutant and 1p19q codeleted [oligodendroglial type]	Diffuse paediatric-type high-grade glioma, RTK1 subtype
Subclass	Glioblastoma, IDH-wt, RTK1 subtype	Oligodendroglioma, IDH-mutant and 1p/19q-codeleted	Diffuse paediatric-type high-grade glioma, RTK1 subtype, subclass A

**Table 2:** A summary of the benefits of methylation-based classification in diffuse gliomas. FFPE—formalin-fixed paraffin-embedded.

Benefits of DNA Methylation Profiling
Provide accurate/correct user-independent diagnosis. Detect diagnostic chromosomal aberrations (e.g., 1p/19q codeletion) with CNA plots. Detect diagnostic mutation status (e.g., IDH) through altered methylation profiles. Predict methylation status of therapy predictive biomarkers (e.g., *MGMT*). Targeted treatment/management according to subclass. Identify novel tumour types and subclasses. Can be used with FFPE tissue and low amounts of DNA/tissue.

**Table 3:** Three examples of histopathological and methylation-based classification. Case 1 is successfully classified by methylation above the threshold (0.9) with the same type as the histopathological diagnosis. Case 2, on the other hand, has a calibrated score just below the threshold. However, as the methylation-based diagnosis is the same as the histopathological diagnosis and supported by the 1p/19q codeletion in the CNA plot, the results are still useful and most likely true. Case 3 is a glioblastoma by histopathology (and a large tumour visible by radiological imaging), but is weakly indicated (calibrated score 0.35) as control tissue (i.e., non-tumour tissue) by methylation classification. Given the low calibrated score, low tumour purity and lack of CNA, the tissue sample used for DNA extraction is not representative of the tumour and, consequently, neither is the methylation-based classification.

	Case 1	Case 2	Case 3
Histopathological diagnosis	Oligodendroglioma, IDH-mutant and 1p/19q-codeleted grade 3	Oligodendroglioma, IDH-mutant and 1p/19q-codeleted grade 3	Glioblastoma, IDH wildtype grade 4
Methylation-based subclass	Oligodendroglioma, IDH-mutant and 1p/19q-codeleted	Oligodendroglioma, IDH-mutant and 1p/19q-codeleted	Control tissue, cerebral hemisphere
Calibrated score	0.91	0.82	0.35
Tumour cell content (%)	72	65	17
Copy-number alterations (CNA)	1p/19q codeletion	1p/19q codeletion	No discernable CNA
